# Illustration of Some Surgical Phenomena by the Theory of Projectiles

**Published:** 1865-01

**Authors:** E. C. Bidwell

**Affiliations:** Surgeon 31st Mass. Vols.


### ILLUSTRATION OF SOME SURGICAL PHENOMENA BY THE THEORY OF PROJECTILES.

By E. C. BIDWELL, Surgeon 31st Mass. Vols.

I suppose that every missile projected from the mouth of a firearm, of whatever kind or calibre, has, in addition to its projectile motion, a motion of rotation upon an axis of its own. If this rotary motion be not the purposed result of the construction of the weapon, it is acquired from accidental causes, which may operate upon the missile at its start or during its flight. Any defect in the gun, or even a slight inequality in the shot, may cause it. Indeed, it is the design of the spiral groove which is introduced into most modern arms, large and small, to give the shot a rotation upon an axis corresponding to the line of traject, thereby to forestall the accidental rotation, which is more likely to be in some other direction, and tends to deflect it from its intended course. This rests upon a manifestation of the physical property of inertia, by which a body in rotation tends to continue to rotate in the same direction, unchanged by any motions that may be impressed upon it as a whole. In other words, the direction of its axis continues parallel to that with which it commences, so long as the rotation continues. This principle is illustrated in the familiar toy, the top, in the spinning plates of the mountebank, and in the revolutions of the heavenly bodies. Now, as the line of flight is always a curve, the axis of rotation ceases to correspond exactly with that line the moment the flight commences. With every succeeding instant the one departs from the other more and more widely. A conical ball from a rifled gun must therefore impinge obliquely by its rotating side, and the more so as the gun is more elevated for long range. That is the theoretical result. I presume that in artillery it is the actual result also. It is said to have had a curious exemplification in the case of the percussion shells sent into Charleston by the Swamp Batteries, which failed to explode because they did not impinge by their apices. In the case of small arms it is found that the designed rotation is often merged in that which proceeds from accidental causes. The impressions upon conical leaden balls picked up on battle-fields, and their positions in various lodging places, prove that they impinge in all directions, and scarcely more frequently by the apex of the cone than by any other part. The acquired rotation of round shot is probably always upon an axis perpendicular, or nearly so, to their projectile direction.

This rotation of round musket balls is probably an important element in determining the erratic course which such balls sometimes take in the body. For it is evident that a ball in rapid rotation entering a medium in which it meets with considerable resistance, will most easily overcome that resistance on that side of which the surface motion is towards the resisting medium, because on that side the force of rotation is added to that of projection, and on the other side is so much deducted from the same force. It will consequently be deflected towards the side of least resultant force. It is well enough to remember that the rotary motion, meeting less resistance than projectile motion from the air, and none from gravity, may continue rapid and even violent after the latter is nearly or quite spent.

Another way in which the rotary motion of projectiles may modify surgical results, is found in the occasional violence of spent cannon balls. A round ball may be moving slowly along the ground, apparently just ready to stop. A careless observer puts his foot against it, and receives an injury which may prove fatal to the member. It has been suggested in explanation of such cases, that the velocity was really much greater than it seemed; but if it were possible to note its progress and direction within such limits of distance that one could put himself in the way of it, the velocity must really have been very moderate. Others have referred to the great momentum which the missile *had*, as if momentum was not exactly proportionate to the velocity, and exhausted when motion ceases. But it is possible for a cannon ball, as for a billiard ball, or a marble, to have a rapid rotary motion after its projectile motion has ceased, sufficient to make it a dangerous neighbor. In that condition anything not capable of greater resistance than the human body, brought into forcible opposition with it, would inevitably suffer violence, precisely as it would if brought into like contact with machinery in like rapid and irresistible motion.

The same principle comes in to modify the injury in oblique impingement by cannon balls. If the rotation is such that on the side impinging the surface is moving forward, or in approximately the same direction as the projectile motion, a laceration results, co-extensive with its touch. The revolving surface moving with the velocity of rotation added to that of projection, *by friction* tears its way through the tissues as in the case already cited. If the rotation be in the contrary direction, so that the rotary motion of the impinging surface in a measure offsets or counterbalances the projectile motion, as in the case of a cannon ball simply rolling over a part, its surface friction may be so slight as to leave the skin unbroken, even unabraded, at the same time that the projectile momentum is sufficient to crush and comminute the parts beneath it to a fatal degree. Of this kind are the cases in which persons are supposed to be knocked down or killed by the "wind" of a cannon ball—an idea which is sufficiently refuted by the fact that shot not only come very near, but into actual collision, carrying away a hat or an arm, without any such stunning or destructive "wind."

It is indisputable that injuries from cannon shot are met with, of these two kinds, in one of which there is profound lesion, with little or no external indication; and in the other frightful laceration, displaying at a glance the full extent of the injury, yet altogether, perhaps, a much less grave injury than the other. That the rotary motion which these projectiles have is sufficient cause to account for the difference of effects, is demonstrable on the principle of mechanics. It is not unimportant to know that something more than mere inexplicable caprice governs movements and determines results, here as elsewhere; that effects follow causes, even in the erratic movements of the missiles of warlike engines.—*Boston Med. and Surg. Journal.*

